# Low-Density Lipoprotein Receptor-Related Protein 6 (LRP6) Is a Novel Nutritional Therapeutic Target for Hyperlipidemia, Non-Alcoholic Fatty Liver Disease, and Atherosclerosis

**DOI:** 10.3390/nu7064453

**Published:** 2015-06-03

**Authors:** Gwang-woong Go

**Affiliations:** Department of Food and Nutrition, Kookmin University, Seoul 136-702, Korea; E-Mail: gwgo@kookmin.ac.kr; Tel.: +82-2-910-5780; Fax: +82-2-910-5249

**Keywords:** LRP6, dyslipidemia, non-alcoholic fatty liver disease, atherosclerosis

## Abstract

Low-density lipoprotein receptor-related protein 6 (LRP6) is a member of the low-density lipoprotein receptor family and has a unique structure, which facilitates its multiple functions as a co-receptor for Wnt/β-catenin signaling and as a ligand receptor for endocytosis. The role LRP6 plays in metabolic regulation, specifically in the nutrient-sensing pathway, has recently garnered considerable interest. Patients carrying an *LRP6* mutation exhibit elevated levels of LDL cholesterol, triglycerides, and fasting glucose, which cooperatively constitute the risk factors of metabolic syndrome and atherosclerosis. Since the discovery of this mutation, the general role of LRP6 in lipid homeostasis, glucose metabolism, and atherosclerosis has been thoroughly researched. These studies have demonstrated that LRP6 plays a role in LDL receptor-mediated LDL uptake. In addition, when the LRP6 mutant impaired Wnt-LRP6 signaling, hyperlipidemia, non-alcoholic fatty liver disease, and atherosclerosis developed. LRP6 regulates lipid homeostasis and body fat mass via the nutrient-sensing mechanistic target of the rapamycin (mTOR) pathway. Furthermore, the mutant LRP6 triggers atherosclerosis by activating platelet-derived growth factor (PDGF)-dependent vascular smooth muscle cell differentiation. This review highlights the exceptional opportunities to study the pathophysiologic contributions of LRP6 to metabolic syndrome and cardiovascular diseases, which implicate LRP6 as a latent regulator of lipid metabolism and a novel therapeutic target for nutritional intervention.

## 1. Introduction to LRP6

**Figure 1:**
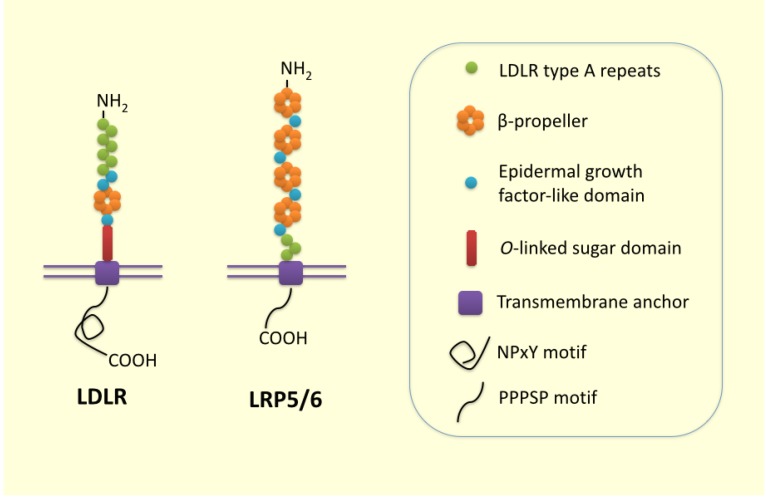
*Structure of low-density lipoprotein receptor (LDLR) and low-density lipoprotein receptor-related protein 6 (LRP6).* LDLR is the patriarch of the LDLR family members, which include LRP6. LDLR and LRP6, and share structurally common motifs: LDLR type A repeats (responsible for binding of ligands), an epidermal growth factor (EGF)-like domain (involved in pH-dependent release of ligands in the endosome), a transmembrane anchor, and a cytoplasmic domain (binding of NPxY and ARH mediates clustering of the receptors into the clathrin-coated pit). Only LRP6 has a PPPSP motif in its cytoplasmic domain.

Low-density lipoprotein receptor-related protein 6 (LRP6) is a member of the low-density lipoprotein receptor (LDLR) family with distinctive structure and ligand-binding functions, which plays a crucial role in lipoprotein endocytosis and in Wnt/β-catenin signaling as a co-receptor. The roles of LRP6 in cell differentiation, proliferation, and migration during embryonic development and in the pathogenesis of different cancer types have been extensively investigated. LRP6 is expressed in most human tissues and is composed of three distinctive cell-surface protein domains, including (1) LDL receptor (LDLR) type A repeats; (2) an epidermal growth factor (EGF)-like domain; and (3) a YWTD (Tyr-Trp-Thr-Asp)-type β propeller domain (Figure 1). The extracellular region of LRP6 includes three clusters of ligand-binding repeats for distinct ligands; namely, Wnt, Dickkopf-related protein 1 (DKK1), and lipoprotein particles. The cytoplasmic C-terminal domain of LRP6 contains at least one copy of a PPPSP (Pro-Pro- Pro-Ser-Pro) motif in place of the standard NPxY (Asn-Pro-any amino acid (x)-Tyr) motif from other LDLR family members. The canonical Wnt signaling pathway consists of cascades of events that follow the binding of Wnt proteins to their receptor, frizzled, and co-receptor, LRP5/6, activating the dishevelled protein family and inhibiting a protein complex that includes axin, glycogen synthase kinase (GSK)-3β, and adenomatous polyposis coli [1]. In addition, activated LRP6 directly inhibits β-catenin phosphorylation by GSK-3β [2]. These events stabilize the cytoplasmic pool of β-catenin and enhance its translocation to the nucleus to interact with other transcriptional regulators, including T-cell factor/lymphoid enhancer factor, triggering the expression of a variety of target genes for the regulation of gluconeogenesis, insulin secretion, and signaling [3,4,5].

Because the initial discovery that LRP6 plays a pivotal role in metabolic syndrome and atherosclerosis, it has become a major focus of scientific investigations on hyperlipidemia, non-alcoholic fatty liver disease, and coronary artery disease (CAD), as well as their associated risk factors (Table 1). Numerous basic and clinical studies have highlighted the key role of Wnt, the receptor LRP6, and its transcriptional cofactor, T-cell transcription factor 4 (TCF4), in the regulation of plasma LDL, triglyceride (TG)/very low-density lipoprotein (VLDL) synthesis, glucose homeostasis, and vascular cell proliferation. Common genetic variants of *LRP6* and *TCF4* [6,7] have been associated with the risks for hyperlipidemia [8,9], atherosclerosis [10], and diabetes [5,11,12,13] in the general population. Minor alleles of the gene encoding the canonical Wnt transcription factor *TCF4* have been associated with reduced expression of TCF4 and hypertriglyceridemia among relatives with familial combined hyperlipidemia [9,14]. However, nutritional intervention of LRP6 functions has not been explored even though it has the potential to provide exceptional insight into previously unidentified disease pathways. Hence, this article aims to demonstrate the value of developing a nutritional intervention strategy for LRP6 by examining its function and underlying pathophysiology.

## 2. Impaired Function of Mutant LRP6

**Figure 2:**
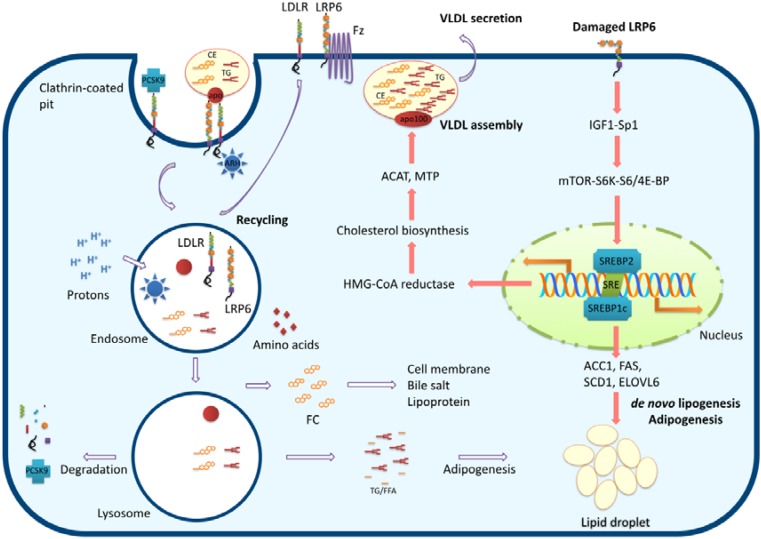
*Hepatic lipid homeostasis is regulated by LRP6.* Recognition of apolipoprotein by the receptor at neutral pH initiates the internalization of ligands, followed by distribution to endosomes. Released ligand particles travel further to lysosomes, where digestive enzymes degrade the ligands. When LRP6 signaling malfunctions, the IGF1-Sp1-mTOR-SREBP1/2 pathway is stimulated. Activation of SREBP1c (required for *de novo* lipogenesis and adipogenesis) and SREBP-2 (which activates cholesterol and LDLR biosynthesis) boost adipogenesis and VLDL assembly, resulting in hyperlipidemia and non-alcoholic fatty liver diseases. CE, cholesterol ester; TG, triglyceride; FFA, free fatty acid; ARH, autosomal recessive hypercholesterolemia protein; PCSK9, proprotein convertase subtilisin/kexin type 9; SREBP, sterol regulatory element-binding protein; Fz, frizzled protein. IGF1, insulin-like growth factor 1; Sp1, specificity protein 1; mTOR, mechanistic target of rapamycin; ACC1, acetyl CoA carboxylase; FAS, fatty acid synthase; SCD1, stearoyl CoA desaturase; ELOVL6, elongation of long-chain fatty acids family member 6; ACAT, acetyl-Co A acetyltransferase; MTP, microsomal triglyceride transfer protein.

Considerable discoveries have been made from the analysis of LRP6 ligand-binding mutants, which result in dysfunctional cell signaling and metabolism. *Ex vivo* examination of primary cells from mutant *LRP6* carriers showed that an arginine to cysteine mutation at residue 611 (R611C) impairs LRP6 phosphorylation by Wnt and inhibits nuclear localization of both β-catenin and TCF4. Moreover, overexpression of β-catenin and TCF4 completely rescued the Wnt signaling cascade in these mutant studies [15,16,17]. Structural analysis of R611C LRP6 reveal that the mechanism of inactivation for this mutation is the disruption of a salt bridge between the substituted arginine at position 611 and an asparagine residue at position 477 [18]. In addition, recent studies have also interpreted a role for LRP6 in the inhibition of non-canonical Wnt signaling, as the smaller heart of *LRP6*-deficient mice are primarily caused by the activation of this non-canonical pathway [19]. Wnt modulates the nutrient-sensing mechanistic target of the rapamycin (mTOR) pathway via the specificity protein 1 (Sp1)-insulin-like growth factor 1 (IGF1) pathway that leads to increased expression of sterol regulatory element-binding protein 1 (SREBP1) and to enhancement of the lipogenic pathway [20] (Figure 2). Discovery of this disease-related *LRP6* allele and its link to altered canonical Wnt signaling has provided novel risk factors and the development of advanced therapeutics for cardiovascular disease.

Unique roles for LRP6 mutations in the regulation of metabolism have recently been established. For instance, a missense mutation (R611C) in human *LRP6* that results in a change of a highly conserved residue in the EGF domain has been identified as the cause of autosomal dominant early onset CAD and metabolic syndrome traits, including hyperlipidemia, type 2 diabetes, osteoporosis, and hypertension [3]. Similarly, common genetic variations within *LRP6* have been associated with increased plasma LDL levels, implicating LRP6 as a potential regulator of lipid metabolism [8,10,17,21]. Furthermore, low plasma levels of Wnt1 and high plasma levels of the LRP6 antagonists sclerostin and DKK1 have been associated with the risks for hyperlipidemia and atherosclerosis [22,23,24]. Other studies have further uncovered the specific role of LRP6 in the regulation of vesicular LDL uptake [17,21]; hepatic lipogenesis and VLDL secretion [20]; glucose homeostasis [15,25]; and vascular smooth muscle cell (VSMC) growth [16] *in vivo* and *in vitro*.

**Table 1:** Summary of the disorders/diseases caused by malfunction of LRP6.

Disorders/Diseases	Phenotypes	Pathway	Reference
**Hypertriglyceridemia**	Increased serum TG levels Increased TG synthesis and VLDL secretion	IGF1-AKT-mTOR-SREBP1	[3,20]
**Hypercholesterolemia**	Decreased LDL clearance in liver and peripheral tissues Increased hepatic synthesis of cholesterol and serum LDL levels	Insig1-SREBP2-HMGCR	[17,21]
**Non-alcoholic fatty liver disease**	Increased lipid/TG accumulation in liver Increased hepatic *de novo* lipogenesis and TG synthesis	MTP-apoB	[20]
**Atherosclerosis**	Early onset of atherosclerosis Increase of lesions and plagues in aorta	PDGF TCF4	[3,16]

## 3. LRP6 and Hyperlipidemia

### 3.1. LRP6 Conducts Cholesterol Homeostasis

Hyperlipidemia refers to a state in which blood has abnormally high levels of lipids, including cholesterol and TG, and is a major risk factor for CAD, the leading cause of death worldwide [26]. The most common type of hyperlipidemia is associated with elevated serum LDL and TG in VLDL [27]. Intensive research aiming to understand the causes of elevated LDL and its mechanisms has led to the discovery of the physiological pathways that regulate LDLR-modulated LDL clearance [28,29,30,31,32,33,34]. More recently, it has been determined that proper LRP6 function is essential for normal LDL clearance [17,21] by forming a complex with the autosomal recessive hypercholesterolemia (ARH) protein and clathrin. Furthermore, co-localization and complex formation between LRP6 and LDLR in LRP6 mutant fibroblast showed impaired internalization of LDL particles. This complex augments cellular LDL binding and uptake both in an LDLR-dependent and LDLR-independent manner [17].

It is also known that mutation of *LRP6* results in reduced LRP6 expression and impaired LDL clearance. Splenic macrophages of *Lrp6^+/−^* mice exhibited decreased LDL clearance compared to that observed in the splenic macrophages of wild-type mice. Skin fibroblasts and B-lymphocytes of *LRP6* mutants showed remarkably impaired LDL internalization, despite having similar affinities for apo B at neutral pH [21]. Studies of LDL clearance in the skin fibroblasts and hematopoietic cells of *LRP6* mutation carriers led to the discovery of the critical role of LRP6 in LDLR-mediated LDL clearance. Neither Wnt stimulation nor Wnt inhibition had any noticeable effect on this process [17,21]. These findings were confirmed independently by a targeted RNAi screening, which identified LRP6 as one of the major regulators of LDL uptake [35,36].

In addition to impaired LDL clearance by LRP6 mutants, cholesterol biosynthesis was severely affected by malfunctioning LRP6. Incorporation of radioisotope-labeled fatty acids into free cholesterol and cholesterol esters was much greater in primary hepatocytes from *Ldlr^−/−^* × *Lrp6_R611C_* compared to those from *Ldlr* knockout mice alone. The mRNA and protein expression levels of the key regulatory enzymes/proteins in cholesterol biosynthesis, including 3-hydroxy-3-methyl-glutaryl-CoA reductase (well known as HMGCR, the target of statins), acetyl-CoA acetyltransferase, and microsomal triglyceride transfer protein, were significantly higher in *Lrp6* mutation carriers [20].

### 3.2. Malfunctioning LRP6 Enhances Hepatic Lipogenesis via the Nutrient-Sensing Pathway

The magnitude of the observed change in LDL clearance due to malfunctions of mutant LRP6 does not sufficiently explain the severity of hyperlipidemia in mutation carriers. Because the liver is the main organ for lipid synthesis and the assembly of lipoproteins, it was postulated that LRP6 might affect hepatic lipogenesis and/or cholesterol synthesis, and subsequently the assembly and secretion of VLDL. Thus, understanding of the mechanisms that regulate TG, cholesterol, and lipoprotein synthesis is of great importance for development of novel biomarkers and drug targets.

Lipid homeostasis in the liver is strongly mediated by components of nutrient-sensing pathways such as mTOR, which is a conserved serine/threonine kinase that regulates multiple anabolic and catabolic pathways. Activation of mTORC1 signaling is strongly associated with increased lipid synthesis and non-alcoholic hepatic steatosis [37]. In addition, stimulation of mTOR pathways promotes insulin-dependent transcription of the stearoyl CoA desaturase 1 (*SCD1*) gene, a critical enzyme that catalyzes the synthesis of monounsaturated fatty acids and controls hepatic lipogenesis and lipid oxidation [38]. According to Ntambi [39], hepatic specific SCD1 knockout protected mice from carbohydrate-induced adiposity and hepatic steatosis. Upon activation by insulin, protein kinase b (PKB, also known as AKT) phosphorylates and inhibits tuberous sclerosis complex 2 (TSC2), a suppressor of mTORC1 [40]. This leads to activation of the mTORC1 downstream peptides S6, S6K, and SREBP. Disruption of the TSC1-TSC2 complex similarly promotes SREBP-dependent lipid synthesis [41].

Investigation of the relationship between LRP6 and lipid synthesis in the liver was accomplished using a human *LRP6* mutation-carrying mouse model. *Lrp6* mutant mice (*Lrp6_R611C_*) had elevated TG and cholesterol synthesis, resulting in lipid accumulation in their livers from the IGF1-AKT-mTOR-SREBP1/2 pathway [20]. Nile red staining assays revealed a greater number of neutral lipids and enzymatic assays were used to determine higher TG and cholesterol ester content in the livers of *Lrp6* mutant mice than in the livers of wild-type mice. Notably, lipogenic enzymes, including acetyl CoA carboxylase, fatty acid synthase, SCD1, diglyceride acyltransferase 1, and elongation of long-chain fatty acids (ELOVL) family member 6, were activated in the livers of *Lrp6* mutant mice. Hepatic SREBP1c and SREBP2 regulate these proteins, and, consequently, both were found to be activated to a significantly higher degree in *Lrp6* mutant mice than in controls. Correspondingly, the SREBP transcription factors are activated by the nutrient-sensing pathway components mTOR and liver X receptor (LXR). As a result, both the activity of mTOR and the expression levels of LXR were elevated in the livers of *Lrp6_R611C_* mice compared to the corresponding values recorded in the livers of wild-type mice. Activation of the mTOR pathway is clearly explained by an augmented IGF1/AKT/mTORC1/SREBP1c cascade. Both mTORC2 and LXR were activated by IGF1 and other related growth factors. IGF1 and its receptor IGF1R are both expressed at much higher levels in the livers of *Lrp6* mutant mice than in the livers of wild-type controls. Furthermore, impaired LRP6 function not only enhanced hepatic lipogenesis but also increased lipid accumulation in the liver and the levels of the different parameters analyzed in liver function tests, such as plasma aspartate aminotransferase and bilirubin [20]. In summary, the unique role of LRP6 in hepatic lipogenesis and hyperlipidemia has been well established. Furthermore, the communication between LRP6 and nutrient-sensing pathways in particular cholesterol sensing pathway and/or mTOR pathway should be a focal point for novel nutritional interventions against hyperlipidemia and non-alcoholic fatty liver disease.

## 4. LRP6 and the Onset of Atherosclerosis

Current knowledge of the mechanisms underlying coronary artery disease (CAD) is largely derived from animal models of severe hypercholesterolemia, using *Ldlr*^−/−^, *ApoE*^−/−^, or *Arh*^−/−^ mice [31,42,43,44]. The investigation of disease pathways in these models has provided significant insights into the pathogenesis of atherosclerosis and the role of inflammation. Serum LDL cholesterol levels of most patients with CAD are, however, only modestly elevated and, for a vast majority, are similar to those of average control populations. The underlying predisposing factors for CAD are widely unknown for the general population. With the advent of modern molecular genetics and an increase in the ability to identify human disease genes, the opportunity for investigation of disease mechanisms in animal models has vastly increased [45,46]. In turn, the identification of *LRP6* has provided an opportunity to identify novel disease pathways [3]. Contrary to standard atherosclerosis, the development of CAD in *LRP6_R611C_* carriers lacks significant contributions from inflammatory processes. *LRP6_R611C_* carriers have normal levels of plasma tumor necrosis factor, interleukin-6, C-reactive protein, apolipoprotein (a), and homocysteine. Computerized tomographic angiography studies of young and healthy mutation carriers reveal diffuse CAD with minimal coronary artery calcification. These findings implicate unique disease mechanisms that are independent of established risk factors and have not been previously studied.

After the initial discovery of the *LRP6* mutation, the role of LRP6 in CAD and atherosclerosis has rapidly begun to investigate. Reduced plasma levels of Wnt1 and elevated levels of LRP6 antagonists have been reproducibly associated with the risk for CAD in patients with early onset CAD, diabetes, and hyperlipidemia in several large clinical studies [8,47]. In addition, common variants of *LRP6* I1062V have been associated with the risk for high LDL cholesterol and CAD in the general population [10]. Accordingly, mice heterozygous or homozygous for the *Lrp6_R611C_* fed with the standard chow diet developed significant aortic wall thickening from proliferation of undifferentiated VSMCS. When fed a high cholesterol diet, these mice developed severe coronary artery and aortic intimal hyperplasia and post carotid injury neo-intima. Furthermore, homozygous *Lrp6_R611C_* and heterozygous crossbred with *Ldlr*^−/−^ mice developed a fulminant proliferative and obstructive CAD, with minimal inflammatory cell contribution. The *Lrp6_R611C_* mouse is an explicit model of a human disease mutation; the mutant *R611C* allele similarly reduces expression of nuclear TCF and β-catenin and impairs Wnt signaling in all examined organs and tissues. Strikingly, many of the vascular phenotypes of the *Lrp6_R611C_* mouse normalize after systemic administration of Wnt3a [48].

*In vitro* investigations have demonstrated that LRP6 binds and ubiquitinates platelet-derived growth factor (PDGF) receptors, while mutant LRP6 enhances their expression and acts as a dominant negative allele product [16]. These findings suggest that the upregulation of LRP6 in coronary artery lesions is likely a compensatory mechanism to inhibit PDGF signaling. PDGF is a known mitogen of mesenchymal stem cells and is a key regulator of phenotypic modulation of VSMCS. Heterozygous and homozygous *LRP6_R611C_* mice replicate many phenotypes of human mutation carriers (CAD, high serum TG, late onset hypertension, and insulin resistance) [48]. Since the metabolic phenotypes and CAD of *LRP6_R611C_* mutation carriers resemble those of the general population, this research is of great importance to aid in the elucidation of novel disease pathways for CAD and to counter pathophysiological conditions by using nutritional intervention.

## 5. Conclusions

CAD accompanied by dyslipidemia remains the single largest cause of death worldwide. Epidemiological studies have established the key roles of several risk factors for CAD, and considerable work has been done to define the underlying molecular basis of these recognized physiological risk factors. Among the most significant advances in this field in recent years is the integration of studies for the identification of genes and pathways that contribute to disease characteristics. Impaired function of LRP6 presents with elevated serum LDL and TG and fasting glucose levels, which together constitute the major components of metabolic syndrome and are major risk factors for atherosclerosis and diabetes. These findings have greatly advanced the understanding of disease pathogenesis and strongly promote the use of nutritional approaches to identify underlying disease-contributing pathways. Undoubtedly, nutritional approaches have the capacity to reveal new physiological pathways that contribute vastly to unrecognized disease pathogeneses, potentially opening entirely new avenues to nutritional intervention.

## Abbreviations

TG: triglyceride
VLDL: very low-density lipoprotein
LDL: low-density lipoprotein
IGF1: insulin-like growth factor 1
mTOR: mechanistic target of rapamycin
Insig1: insulin induced gene 1
SREBP,: sterol regulatory element-binding protein
MTP: microsomal triglyceride transfer protein
PDGF: Platelet-derived growth factor
TCF4: transcription factor 4
